# Treatment outcomes of hepatectomy and systemic chemotherapy based on oncological resectability criteria for hepatocellular carcinoma

**DOI:** 10.1002/ags3.12893

**Published:** 2024-12-20

**Authors:** Shohei Komatsu, Yoshihiko Yano, Nobuaki Ishihara, Masahiro Kido, Hidetoshi Gon, Kenji Fukushima, Takeshi Urade, Hiroaki Yanagimoto, Hirochika Toyama, Takumi Fukumoto

**Affiliations:** ^1^ Division of Hepato‐Biliary‐Pancreatic Surgery, Department of Surgery Kobe University Graduate School of Medicine Kobe Hyogo Japan; ^2^ Division of Gastroenterology, Department of Internal Medicine Kobe University Graduate School of Medicine Kobe Hyogo Japan

**Keywords:** borderline resectable, hepatectomy, hepatocellular carcinoma, oncological resectability criteria, systemic chemotherapy

## Abstract

**Aim:**

The oncological resectability criteria for hepatocellular carcinoma (HCC) have recently been established (R/BR1/BR2), and validating the outcomes is an urgent issue. This study aimed to analyze the outcomes of hepatectomy and systemic chemotherapy based on the oncological resectability criteria.

**Methods:**

A total of 931 patients in the hepatectomy group and 273 in the systemic chemotherapy group who received atezolizumab/bevacizumab, lenvatinib, or durvalumab plus tremelimumab were recruited.

**Results:**

The median survival times (MST) in the hepatectomy group were R, 107.2 mo; BR1, 44.4 mo; and BR2, 18.4 mo (*p* < 0.0001). The MSTs in the systemic chemotherapy group were R, 16.3 mo; BR1, 24.5 mo; and BR2, 16.1 mo (*p* = 0.3598). A comparison of survival of patients in the BR2 category revealed no significant difference between the two groups for those with modified albumin‐bilirubin grade 1 + 2a (*p* = 0.7343) and grade 2b + 3 (*p* = 0.6589). The BR2 definition comprised three tumor factors, and the MST of patients with only one BR2‐defining factor tended to be better in the hepatectomy group than in the systemic chemotherapy group (22.9 vs 20.2 mo, *p* = 0.0977). Meanwhile, the MST tended to be better in the systemic chemotherapy group than in the hepatectomy group (16.5 vs 12.6 mo) for those with two to three BR2‐defining factors, although the difference was insignificant (*p* = 0.4252).

**Conclusion:**

The oncological resectability criteria for HCC effectively stratified the prognosis after hepatectomy. Treatment outcomes of hepatectomy in patients with two to three BR2‐defining factors are limited, suggesting the need for multidisciplinary treatment.

## Graphical abstract

The oncological resectability criteria for HCC effectively stratified the prognosis after hepatectomy, and the treatment outcomes of hepatectomy for patients in the R and BR1 groups were favorable, indicating the validity of hepatectomy for these patient populations. Meanwhile, treatment outcomes of hepatectomy in patients with two to three BR2‐defining factors are limited, suggesting the need for multidisciplinary treatment.

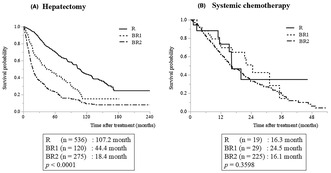

## 1 INTRODUCTION

Hepatocellular carcinoma (HCC) is the most common primary liver cancer subtype and among the leading causes of cancer‐related deaths worldwide.^1^, ^2^ While indications for curative treatments, including hepatectomy, radiofrequency ablation, and liver transplantation, are limited to the early stages of cancer,^3^, ^4^ most cases are diagnosed at an advanced stage, for which systemic chemotherapy is generally indicated. Recently, remarkable advancements have been made in the development of systemic chemotherapies for HCC. In the IMbrave150 trial, atezolizumab plus bevacizumab (Ate/Bev), which combined antiprogrammed death ligand 1 (PD‐L1) and antivascular endothelial growth factor, showed superior survival outcomes and objective response rates compared with sorafenib.^5^ Durvalumab plus tremelimumab (Dur/Tre), which combined anticytotoxic T lymphocyte‐associated antigen 4 and anti‐PD‐L1, showed superiority over sorafenib in overall survival in the HIMALAYA trial.^6^ Accordingly, Ate/Bev and Dur/Tre are recommended as the first‐line treatment for advanced HCC in the Barcelona Clinic Liver Cancer (BCLC) guidelines.^7^ Lenvatinib (LEN) is a multiple‐receptor tyrosine kinase inhibitor that inhibits the vascular endothelial growth factor and fibroblast growth factor.^8^, ^9^, ^10^ The REFLECT trial revealed that, compared to sorafenib, LEN showed favorable outcomes for unresectable HCC^11^; overall survival was comparable between the two groups, while progression‐free survival was significantly better than that of sorafenib. In clinical settings, these three regimens have been frequently used in the early lines owing to their high efficacy.

Recent topics include the oncological resectability criteria for HCC (BR‐HCC criteria), which were established by a Japanese expert consensus in 2023.^12^ In these criteria, the concept of unresectable tumors did not exist; the decision was to classify borderline resectability into two criteria, defined from the therapeutic perspective of hepatectomy. Together with resectability, these criteria can be classified into three categories: resectable (R), borderline resectable 1 (BR1), and borderline resectable 2 (BR2).^12^ However, the clinical outcomes of systemic chemotherapy based on these criteria are unknown. These criteria are defined solely in terms of oncological factors, whereas outcomes including liver function factors should be examined.

The present study aimed to investigate the clinical outcomes of hepatectomy and systemic chemotherapy based on the BR‐HCC criteria and discuss optimal therapeutic approaches to borderline HCC.

## 2 PATIENTS AND METHODS

### 2.1 Patient population

The present study included 931 patients who underwent hepatectomy between January 2000 and December 2022 at Kobe University and 273 patients receiving systemic chemotherapy, including Ate/Bev, LEN, and Dur/Tre, from June 2018 to October 2023, at Kobe University and Kobe Minimally Invasive Cancer Center. All patients underwent pretreatment laboratory blood tests, including viral serology tests and measurements of serum alpha‐fetoprotein, serum protein induced by vitamin K absence or antagonist II, serum albumin, total bilirubin, and prothrombin time. Liver function was assessed using the Child–Pugh status, albumin–bilirubin (ALBI) score,^13^ and modified ALBI (mALBI) grade.^14^ The ALBI grade was calculated based on serum albumin and total bilirubin levels as follows: ALBI score = (0.66 × log10 bilirubin [μmol/L]) + (−0.085 × albumin [g/L]). The mALBI grade was classified into four grades according to the following cutoff values: grade 1, ≤ −2.60; grade 2a, −2.60 < to ≤ −2.27; grade 2b, −2.27 < to ≤ −1.39; and grade 3, > −1.39.^14^, ^15^ HCC diagnosis and tumor characteristics were evaluated using contrast‐enhanced thoracic and abdominal computed tomography, transabdominal ultrasonography, and magnetic resonance imaging. The performance status was assessed according to the Eastern Cooperative Oncology Group criteria, and the BCLC stage was used to determine tumor stages.^7^ The extent of hepatectomy was primarily determined by the future remnant liver volume and liver function predicted from the indocyanine green retention rate at 15 min. This study was approved by the Ethics Committee of Kobe University (B240007) and Kobe Minimally Invasive Cancer Center, and all procedures were conducted in accordance with the ethical guidelines of the 1975 Declaration of Helsinki. All patients provided written informed consent before treatment.

### 2.2 Indication and procedure of hepatectomy

The inclusion criteria of hepatectomy for HCC were as follows: (1) general condition tolerable to surgery, (2) liver function with Child–Pugh class A or B, and (3) estimated remnant liver volume ≥35% after scheduled hepatectomy, calculated based on preoperative imaging volumetry. Hepatectomies were performed using an open or laparoscopic approach. Between January 2000 and October 2011, only open hepatectomies were performed; however, from November 2011, either open or laparoscopic hepatectomies were performed. Open hepatectomy was performed through a right subcostal incision with midline extension, whereas laparoscopic hepatectomy was performed routinely using five trocars. Hepatic transection was performed using an ultrasonic surgical aspirator (CUSA; Cavitron Lasersonic, Stamford, CT, USA) or harmonic scalpel (Ethicon Endo‐Surgery, Cincinnati, OH, USA). The Pringle maneuver was routinely used to occlude blood inflow to the liver in both open and laparoscopic hepatectomies.

### 2.3 Indication and treatment details of systemic chemotherapy

The eligibility criteria of systemic chemotherapy with Ate/Bev, LEN, and Dur/Tre were as follows: (1) Eastern Cooperative Oncology Group performance status of 0–2, (2) HCC without indication for locoregional treatment, and (3) Child–Pugh classes A and B. The ineligibility criteria for these treatments were as follows: (i) history of serious medical conditions, (ii) ascites refractory or minimally responsive to therapy, and (iii) severe autoimmune diseases (this criterion was specific for Ate/Bev and Dur/Tre). The Ate/Bev dosage regimen included intravenous administration of atezolizumab (1200 mg) and bevacizumab (15 mg/kg) every 3 weeks. LEN was orally administered at 8 mg/d to patients with body weight <60 kg and 12 mg/d to those weighing ≥60 kg. The Dur/Tre dosing regimen included the intravenous administration of tremelimumab (300 mg, one dose) plus durvalumab (1500 mg every 4 weeks).

### 2.4 Study design and statistical analyses

**FIGURE 1:**
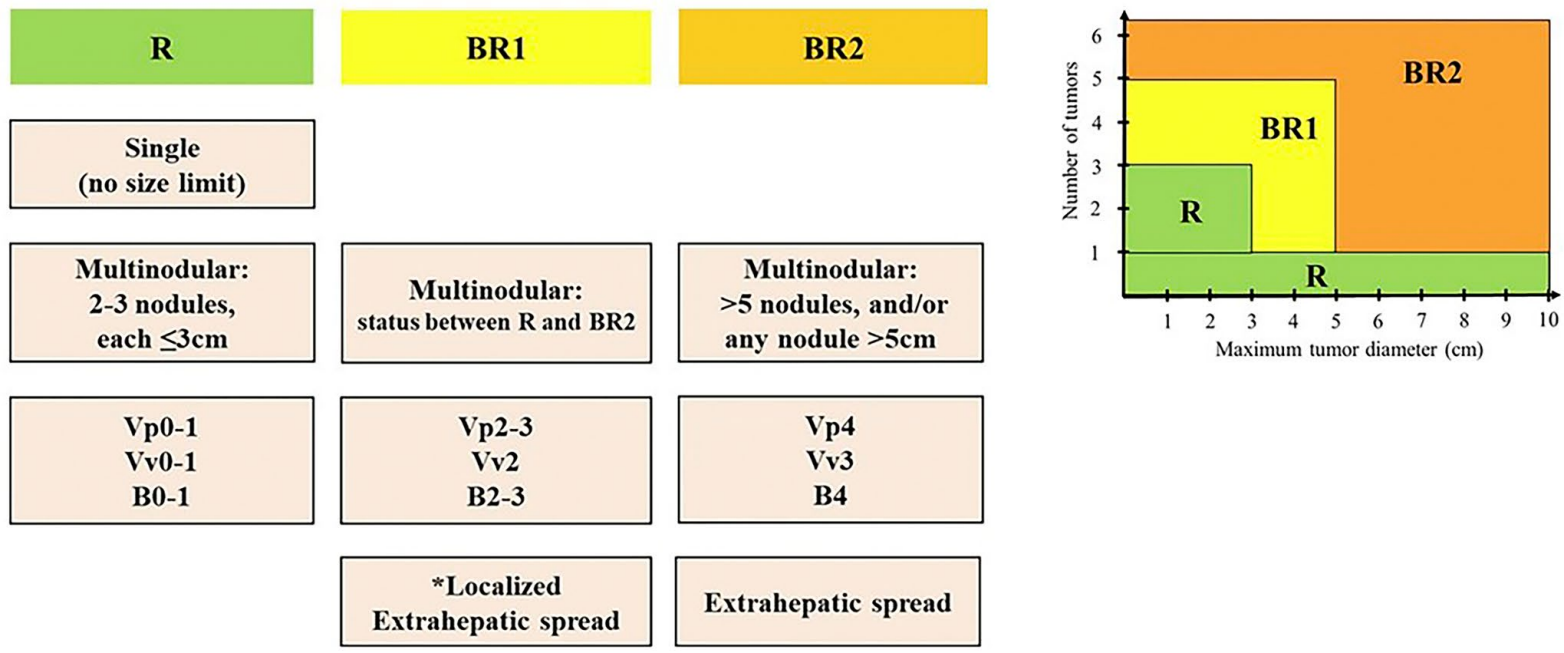
Oncological resectability criteria for hepatocellular carcinoma by Japanese expert consensus 2023. BR1, borderline resectable 1; BR2, borderline resectable 2; R, Resectable. *Examples of localized extrahepatic spread are as follows: solitary nodal involvement at no. 3, 8, or 12 lymph nodes, localized peritoneal dissemination, unilateral adrenal metastasis, or oligometastasis to the lung.

All patients in the hepatectomy and systemic chemotherapy groups were classified into three groups from an oncological viewpoint based on the BR‐HCC criteria: R, BR1, and BR2 (Figure 1). Owing to the small number of patients in the R and BR1 groups in the systemic chemotherapy group, a comparison of outcomes between hepatectomy and systemic chemotherapy was performed in the BR2 category. Liver function was classified into two groups: mALBI grade 1 + 2a (1/2a) and 2b + 3 (2b/3), and the outcomes of the BR2 group were compared between the hepatectomy and systemic chemotherapy groups according to liver function. Furthermore, according to the BR‐HCC criteria, BR2 is dependent on three tumor factors: (1) multiple lesions with more than five nodules or >5 cm in diameter, (2) major vascular invasion (Vp4, Vv3, or B4), and (3) extrahepatic diseases not fulfilling the localized factor classified as BR1 (Figure 1). All patients in the BR2 group who underwent hepatectomy and systemic chemotherapy were checked to see how many of these three factors were obtained, and the outcomes were compared between the two groups: obtaining only one BR2‐defining factor versus obtaining two to three BR2‐defining factors.

**FIGURE 2:**
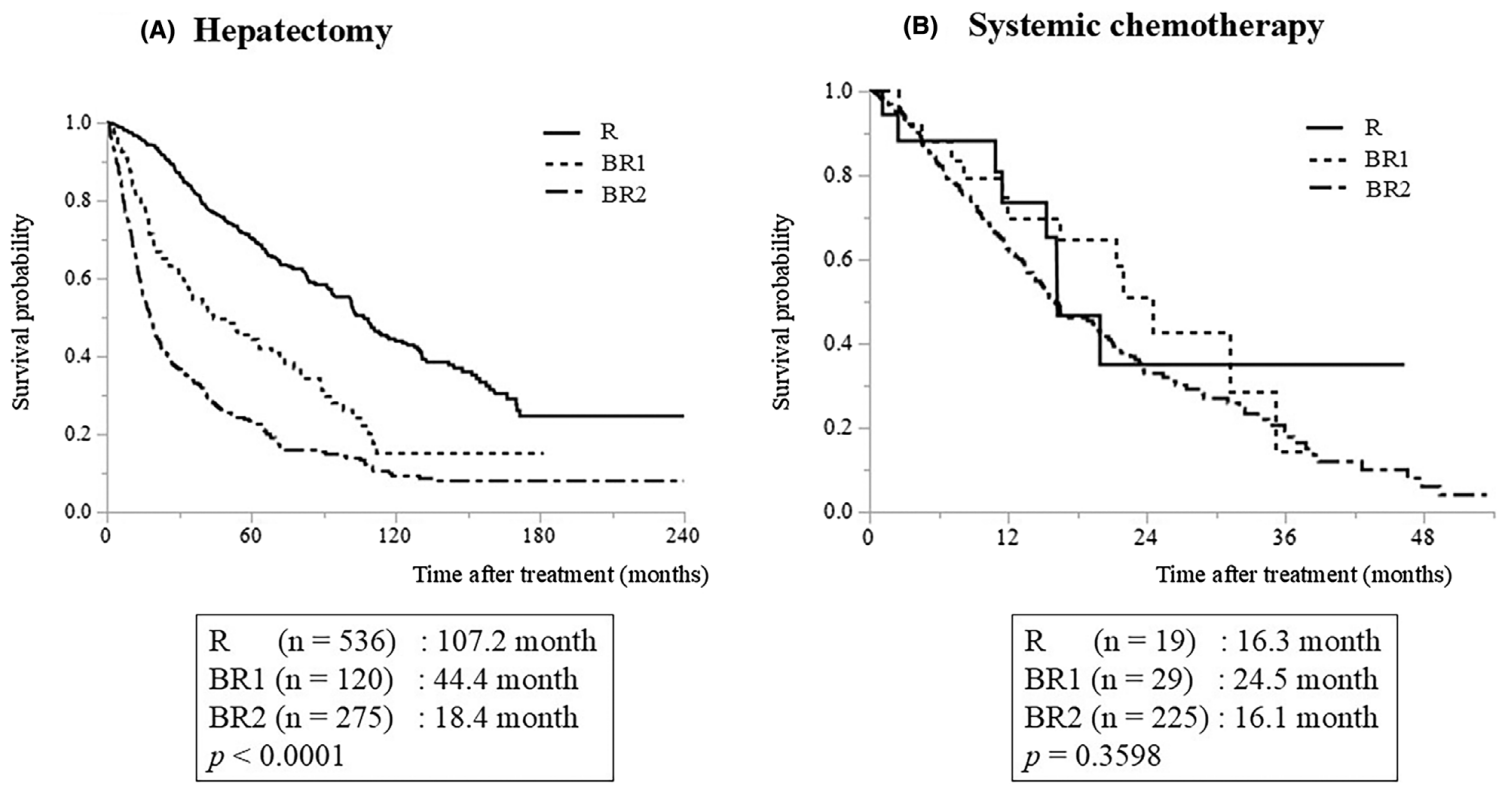
Treatment outcomes according to the oncological resectability criteria: (A) hepatectomy and (B) systemic chemotherapy.

**FIGURE 3:**
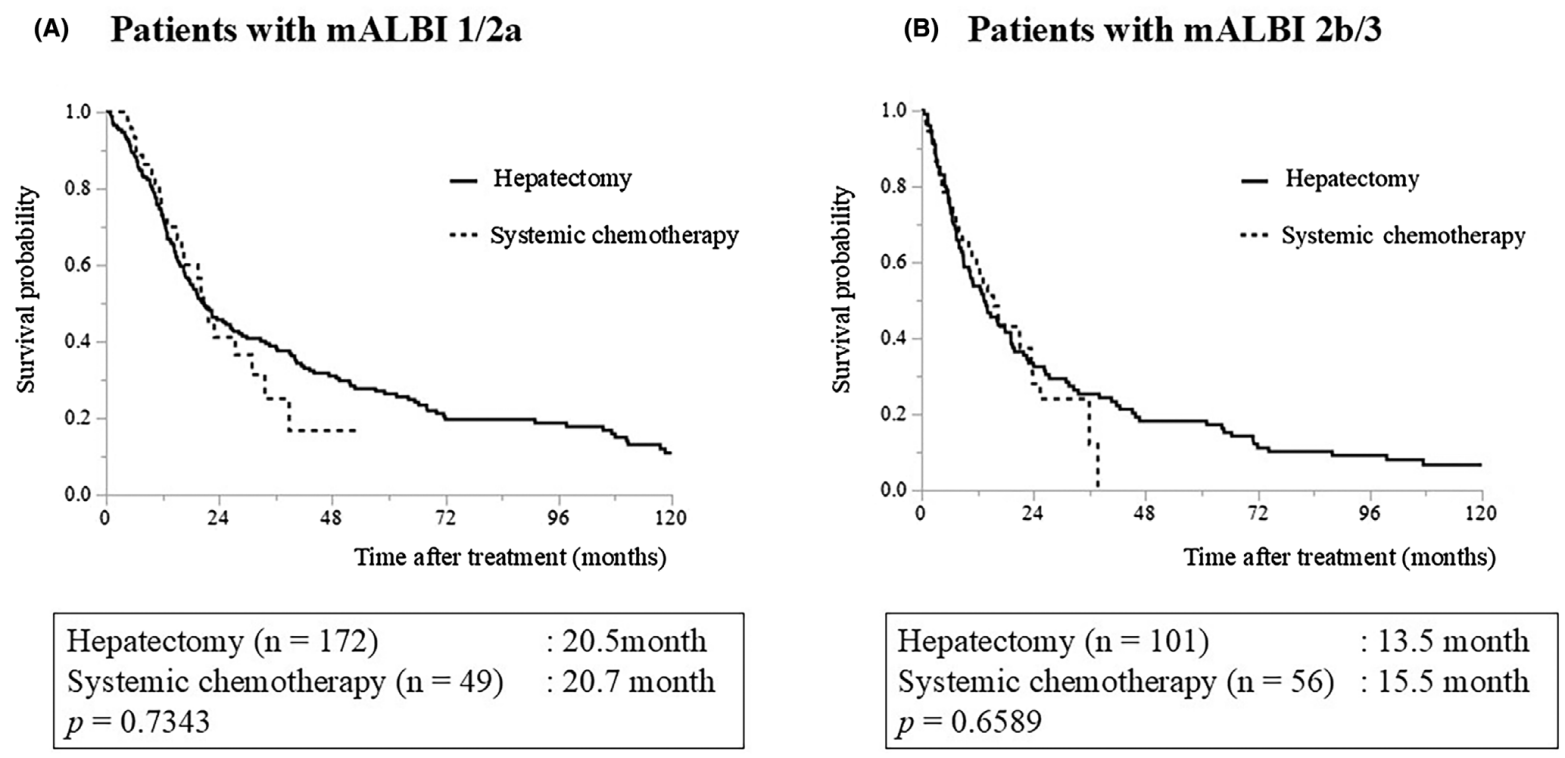
Treatment outcomes of patients in BR2 (hepatectomy versus systemic chemotherapy): (A) Patients with mALBI grade 1 + 2a. (B) Patients with mALBI grade 2b + 3. BR2, borderline resectable 2; mALBI: Modified albumin‐bilirubin.

**FIGURE 4:**
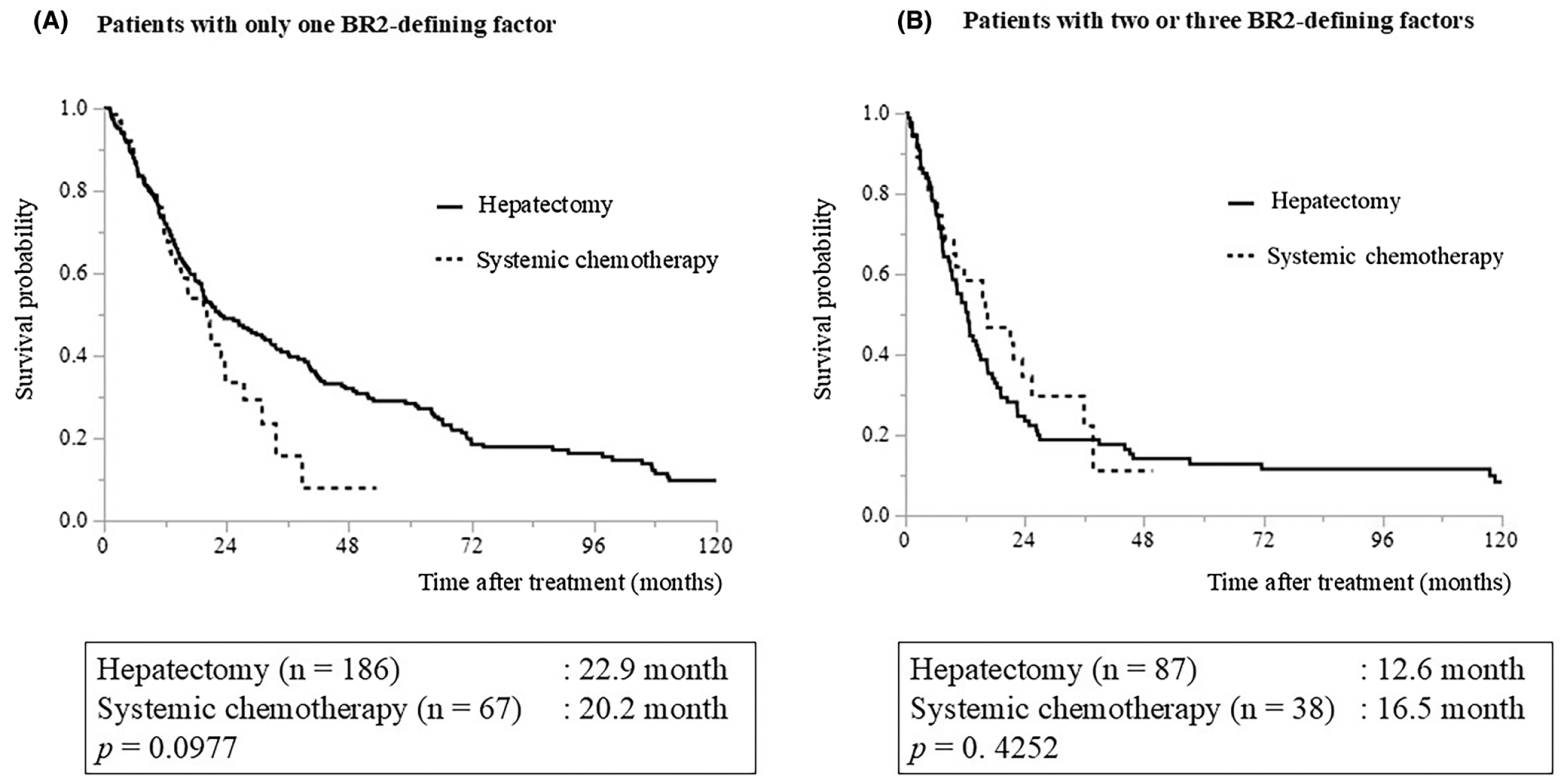
Treatment outcomes of patients in BR2 (hepatectomy versus systemic chemotherapy): (A) Patients with only one BR2‐defining factor. (B) Patients with two to three BR2‐defining factors. BR2, borderline resectable 2.

Differences in baseline characteristics between patients in the two groups were compared using Fisher's exact test or the chi‐square test for categorical variables and Student's *t*‐test or the Mann–Whitney *U* test for continuous variables. Variables not conforming to a normal distribution are expressed as median (range), and those following a normal distribution are expressed as mean ± standard deviation. Overall survival was calculated from the date of hepatectomy or first administration of systemic chemotherapy to the date of death or final observation in the case of censoring and was estimated using the Kaplan–Meier method. Survival analysis, based on the BR‐HCC criteria for hepatectomy and systemic chemotherapy (Figure 2), was performed using all treatment opportunities for each treatment group (with duplicate cases in each treatment group). However, in the comparison of treatment results between hepatectomy and systemic chemotherapy (Figures 3 and 4, Figures S1 and S2), all duplicate cases were deleted and only the group in which each case was initially treated was analyzed. Patients who received systemic chemotherapy for recurrence after initial hepatectomy were not included in the systemic chemotherapy group, but were analyzed only as a hepatectomy group. Patients who received multiple regimens of systemic chemotherapy were analyzed for prognosis only at the time of the first regimen. All statistical analyses were performed using JMP 17 (SAS Institute, Cary, NC, USA). *p* < 0.05 indicated statistical significance.

## 3 RESULTS

### 3.1 Treatment outcomes of all treatment opportunities in each treatment

The baseline background factors of all treatment opportunities in each treatment are shown in Table 1 (there was an overlap of cases by treatment or chemotherapy regimen). A total of 931 patients who underwent hepatectomy and 273 who received systemic chemotherapy were included in this cohort. Systemic chemotherapy regimens include Ate/Bev in 118 (43.2%), LEN in 137 (50.2%), and Dur/Tre in 18 (6.6%). The median patient age was 69 y (range: 21–93 y) in the hepatectomy group and 72 y (range: 34–90 y) in the systemic chemotherapy group. Regarding liver function classification, 689 (74.0%) and 242 (26.0%) patients were classified as mALBI 1/2a and 2b/3, respectively, in the hepatectomy group. In the systemic chemotherapy group, 144 (52.7%) and 129 (47.3%) patients had mALBI grades 1/2a and 2b/3, respectively. According to the BR‐HCC criteria, 536 (57.6%), 120 (12.9%), and 275 (29.5%) patients were classified into R, BR1, and BR2, respectively, in the hepatectomy group. In the systemic chemotherapy group, 19 (7.0%), 29 (10.6%), and 225 (82.4%) patients were classified as R, BR1, and BR2, respectively.

**TABLE 1:** Treatment opportunities with hepatectomy and systemic chemotherapy.

	Hepatectomy (*n* = 931)	Systemic chemotherapy (*n* = 273)
Age (y)^a^	69 (21–93)	72 (34–90)
Sex ratio (M:F)	777:154	218:55
ALBI grade		
1	454 (48.8)	74 (27.1)
2	463 (49.7)	182 (66.7)
3	14 (1.5)	17 (6.2)
mALBI grade		
1 + 2a	689 (74.0)	144 (52.7)
2b + 3	242 (26.0)	129 (47.3)
BR definition		
R	536 (57.6)	19 (7.0)
BR1	120 (12.9)	29 (10.6)
BR2	275 (29.5)	225 (82.4)

*Note*: Values in parentheses are percentages unless indicated otherwise; values are ^a^median (range).

Abbreviations: ALBI, albumin‐bilirubin; BR, borderline; BR1, borderline resectable 1; BR2, borderline resectable 2; mALBI, modified albumin‐bilirubin; R, resectable.

The median survival times (MST) according to the BR‐HCC criteria were R: 107.2, BR1: 44.4, and BR2: 18.4 mo, respectively, in the hepatectomy group, with a significant difference (Figure 2A *p* < 0.0001). The MST, according to the BR‐HCC criteria, were R: 16.3, BR1: 24.5, and BR2: 16.1 mo in the systemic chemotherapy group, respectively, without a significant difference (Figure 2B *p* = 0.3598).

### 3.2 Patient characteristics of hepatectomy and systemic chemotherapy for patients in BR2

The characteristics of all patients who underwent hepatectomy and systemic chemotherapy are reported in Table 2 (all duplicate cases were deleted, and only the group in which each case was treated as the initial treatment was analyzed). The median age was significantly lower in the hepatectomy group than in the systemic chemotherapy group (67 vs 72 y, *p* < 0.0001). Regarding ALBI grade, 101 (37.0%), 164 (60.1%), and 8 (2.9%) patients were classified as ALBI grades 1, 2, and 3, respectively. In the systemic chemotherapy group, 25 (23.8%), 72 (68.6%), and 8 (7.6%) patients were classified as having ALBI grades 1, 2, and 3, respectively (*p* = 0.0134). Regarding the mALBI grade classification, 172 (63.0%) and 101 (37.0%) patients were classified as mALBI grades 1/2a and 2b/3, respectively, in the hepatectomy group and 49 (46.7%) and 56 (53.3%) were classified as mALBI grades 1/2a and 2b/3, respectively, in the systemic chemotherapy group. The proportion of patients with impaired liver function (mALBI grade 2b/3) was significantly higher in the systemic chemotherapy group (*p* = 0.0041). Patients in the BR2 category were further divided into two groups, one or two to three BR2‐defining factors, according to the presence of some of the three factors that fulfill the BR2 definition. There was no significant difference in the proportion of obtaining BR2‐defining factors (one or two to three) between the hepatectomy and systemic chemotherapy groups (*p* = 0.4258).

**TABLE 2:** Patient characteristics of hepatectomy and systemic chemotherapy in BR2.

	Hepatectomy (*n* = 273)	Systemic chemotherapy (*n* = 105)	*p* value
Age (y)^a^	67 (21–91)	72 (43–90)	<0.0001^b^
Sex ratio (M:F)	241:32	86:19	0.1130^b^
ALBI grade			
1	101 (37.0)	25 (23.8)	0.0134
2	164 (60.1)	72 (68.6)	
3	8 (2.9)	8 (7.6)	
mALBI grade			
1 + 2a	172 (63.0)	49 (46.7)	0.0041
2b + 3	101 (37.0)	56 (53.3)	
Obtained BR2 factors			
One factor	186 (68.1)	67 (63.8)	0.4258
Two to three factors	87 (31.9)	38 (36.2)	

*Note*: Values in parentheses are percentages unless indicated otherwise; values are ^a^median (range).

^b^ Student's *t* test.

Abbreviations: ALBI, albumin‐bilirubin; BR2, borderline resectable 2; mALBI, modified albumin‐bilirubin.

### 3.3 Treatment outcomes of BR2: Hepatectomy versus systemic chemotherapy

Regarding patients in the BR2 category (those with mALBI grade 1/2a), the MSTs were 20.5 mo in the hepatectomy group and 20.7 mo in the systemic chemotherapy group, respectively, without a significant difference (Figure 3A, *p* = 0.7343). The MSTs of patients with mALBI grade 2b/3 were 13.5 and 15.5 mo, respectively, without significant difference (Figure 3B, *p* = 0.6589). Regarding the outcomes of patients with only one BR2‐defining factor, the MSTs of hepatectomy and systemic chemotherapy were 22.9 and 20.2 mo, respectively, without significant difference (Figure 4A, *p* = 0.0977). However, the 3‐year overall survival rates were 40.9% and 15.6% in the hepatectomy and systemic chemotherapy groups, respectively. Meanwhile, the MSTs of patients with two to three BR2‐defining factors were 12.6 mo in the hepatectomy group and 16.5 mo in the systemic chemotherapy group without significant difference (Figure 4B, *p* = 0.4252).

Among the patients with only one BR2‐defining factor (*n* = 253: hepatectomy, *n* = 186; systemic chemotherapy, *n* = 67), 151 and 102 patients were categorized as mALBI 1/2a and mALBI 2b/3, respectively. In this population (patients with only one BR2‐defining factor), the MST of patients with mALBI grade 1/2a were 28.9 mo in the hepatectomy group and 20.7 mo in the systemic chemotherapy group, respectively, without significant difference (Figure S1A, *p* = 0.1652). The MST of patients with mALBI grade 2b/3 was 18.0 mo in the hepatectomy group and 14.1 mo in the systemic chemotherapy group, respectively, without significant difference (Figure S1B, *p* = 0.5966).

Among the patients with two to three BR2‐defining factors (*n* = 125: hepatectomy, *n* = 87; systemic chemotherapy, *n* = 38), 70 and 55 patients were categorized as mALBI 1/2a and mALBI 2b/3, respectively. In this population (patients with two to three BR2‐defining factors), the MST of patients with mALBI grade 1/2a was 13.6 mo in the hepatectomy group and 21.7 mo in the systemic chemotherapy group, respectively, without significant difference (Figure S2A, *p* = 0.2464). The MST of patients with mALBI grade 2b/3 was 7.5 mo in the hepatectomy group and 15.5 mo in the systemic chemotherapy group, respectively, without significant difference (Figure S2B, *p* = 0.6980). The MST tended to be better in the hepatectomy group than the systemic chemotherapy group for patients with only one BR‐2 defining factor, regardless of liver function. In patients with two to three BR‐2 defining factors, the MST was better in the systemic chemotherapy group than the hepatectomy group regardless of liver function, though the difference was insignificant.

## 4 DISCUSSION

HCC is a highly heterogeneous tumor, and the indication for hepatectomy for HCC must be considered based on the tumor, liver function, and technical factors; however, no consensus has yet been reached. The definition of unresectable HCC varies significantly among institutions. Although the concept of borderline resectable or conversion surgery was established and developed as a general treatment strategy for pancreatic cancer and metastatic liver cancer,^16^, ^17^ it was not fostered for HCC because of the low effectiveness of systemic chemotherapy. Along with recent drastic advances in systemic chemotherapy, establishing a uniform definition of HCC tumor status is an urgent issue; thus, the recently established oncological resectability criteria for HCC (BR‐HCC criteria) constitute a groundbreaking first step.^12^ The present study demonstrated the effectiveness of the BR‐HCC criteria in stratifying prognosis after hepatectomy; however, the effectiveness of stratifying systemic chemotherapy outcomes requires further investigation.

The MSTs of patients in the hepatectomy group with R and BR1 were 107.2 and 44.4 mo, respectively. These were favorable outcomes, indicating the validity of hepatectomy in patients in the R and BR1 groups. The BR‐HCC criteria define BR1 based on three oncological factors: tumor size and number, macroscopic vascular invasion, and localized extrahepatic metastases (Figure 1). However, based on the BCLC treatment strategy, these tumor statuses were not indicated for hepatectomy, and systemic chemotherapy or transcatheter arterial chemoembolization were recommended.^7^ Several previous studies have demonstrated the effectiveness of hepatectomy for HCC that meets the criteria for BR1.^18^, ^19^ Although detailed prognostic analyses according to liver function or tumor factors within the BR1 criteria are required in a larger number of cases, hepatectomy should be considered first for cases within the BR1 criteria.

The present study focused on comparing the treatment outcomes between hepatectomy and systemic chemotherapy in patients in the BR2 category. There was no significant difference in the survival curves between the hepatectomy and systemic chemotherapy groups, irrespective of liver function (mALBI 1/2a and 2b/3). The MSTs of patients in the BR2 category were ~20 mo in the mALBI 1/2a group and 13–16 mo in the mALBI 2b/3 group for both hepatectomy and systemic chemotherapy (Figure 3). The three BR2‐defining factors of tumor size or number, macroscopic vascular invasion, and extrahepatic metastases (Figure 1) are considered to indicate an advanced tumor status, and the presence of any one of them is generally considered to indicate unresectable tumor. However, some reports suggest the efficacy of hepatectomy in a population with one of the BR2‐defining factors.^20^, ^21^, ^22^ The present study has shown a tendency for favorable outcomes of hepatectomy over systemic chemotherapy for patients in the BR2 category, with only one of the three BR2‐defining factors. In contrast, in cases where two to three BR2‐defining factors were obtained, systemic chemotherapy tended to result in better survival irrespective of liver function, despite the difference being nonsignificant. Although the results of this study should not be relied upon solely because of the large patient selection bias, indications for surgical intervention should be considered in cases with only one BR2‐defining factor. For patients with two to three BR2‐defining factors, the benefits of upfront hepatectomy can be considered minimal, and prompt introduction of systemic chemotherapy is preferable.

Recent advancements in systemic chemotherapy have established the concept of conversion therapy, in which local treatment is introduced after systemic chemotherapy.^23^ Kudo advocates the novel concept named “ABC conversion” comprising Ate/Bev therapy followed by curative conversion therapy.^24^ This groundbreaking concept can potentially revolutionize the treatment strategies for advanced HCC. In addition to hepatectomy, radiofrequency ablation, and transcatheter arterial chemoembolization, the effectiveness of radiotherapy as a local treatment after conversion has recently been discussed.^25^ Establishing the BR‐HCC criteria and spreading the conversion concept may accelerate the trend of administering systemic chemotherapy upfront. However, even with Ate/Bev, LEN, and Dur/Tre administration, which show a high response rate, nearly 20% of cases develop progressive disease. The unintentional introduction of systemic chemotherapy for resectable tumors requires caution because of the risk of tumor progression to unresectable status. Recent advancements in radiotherapy, represented by particle radiotherapy, have led to radiotherapy being considered a curative local treatment for HCC.^26^ Thus, the indication for curative local treatment, including hepatectomy, radiofrequency ablation, and radiotherapy, should be considered before introducing systemic chemotherapy, especially in BR1 cases. Given the recent treatment algorithms for advanced HCC, systemic chemotherapy will become the mainstay for advanced HCC in BR2 cases. However, a certain population would benefit from local treatments even in BR2 cases; thus, the treatment option with an upfront local treatment approach should be considered. Achieving a complete response only with systemic chemotherapy may be impossible; thus, a multidisciplinary approach comprising a combination or conversion concept with systemic chemotherapy and local treatments is essential to achieve a long‐term prognosis. Preserving liver function is equivalent to tumor control in HCC treatment, and the treatment sequence is crucial to achieve this. Further studies are warranted on the sequence of systemic chemotherapy, including regimen order, appropriate timing of introducing local treatments, and local treatment modality.

The main limitations of this study include its retrospective design and review of surgical outcomes at a single institution and systemic chemotherapy outcomes at two institutions. In addition, significant selection bias relative to the institution and patients precludes definite conclusions. BR1 and BR2 are each formed from three tumor factors, and we considered their impact on prognosis to be the same in this study; however, whether this may be the case is a matter of debate. Shindoh et al have reported that each of these factors independently affects prognosis, which could be one rationale for our consideration.^27^ In any case, the study contains many biases; to minimize the bias inherent in a single‐center evaluation, further evaluation in a multicenter study will be required to obtain more robust evidence.

This is the first study to compare the treatment outcomes of hepatectomy and systemic chemotherapy for HCC based on the BR‐HCC criteria. We believe that it provides important insights into treatment selection in the new era of systemic chemotherapy.

## 5 CONCLUSION

The oncological resectability criteria for HCC effectively stratified prognosis after hepatectomy. The treatment outcomes of hepatectomy for patients in the R and BR1 groups were favorable, indicating the validity of hepatectomy for these patient populations. Treatment outcomes of hepatectomy in patients with two to three BR2‐defining factors are limited, suggesting the need for a multidisciplinary treatment approach.

## AUTHOR CONTRIBUTIONS

Shohei Komatsu conceived and designed the study, collected and analyzed the data, and wrote the article. Yoshihiko Yano, Nobuaki Ishihara, and Masahiro Kido collected and interpreted the data. Hidetoshi Gon, Kenji Fukushima, and Takeshi Urade contributed to statistical analysis. Hiroaki Yanagimoto and Hirochika Toyama revised the article. Takumi Fukumoto supervised the study.

## FUNDING INFORMATION

This research did not receive any grants from funding agencies in the public, commercial, or nonprofit sectors.

## CONFLICT OF INTEREST STATEMENT

The authors declare no conflicts of interest for this article.

## ETHICS STATEMENT

Approval of the research protocol: This study conformed to the provisions of the Declarations of Helsinki, and the protocol was approved by the Kobe University Hospital Institutional Review Board (Approval no. B240007) and the Institutional Review Board of Kobe Minimally Invasive Cancer Center.

## Supporting information


Figure S1.

Figure S2.

Data S1.

## APPENDIX A DISCUSSANT

### A.1 Professor Junichi Shindoh

Thanks for the wonderful talk. First, I would like to congratulate you on your intensive work and impressive results of surgical resection for patients with advanced hepatocellular carcinoma. I feel this study is very important because your results highlighted how we should use novel criteria of resectability to look for the optimal candidate for surgery among the patients with advanced hepatocellular carcinoma. To further clarify the importance of the data, I would like to ask you two questions.

First, it is important to look at the prognostication ability of the novel criteria, especially among patients with advanced disease, which is not suitable for surgery. You have mentioned that in your cohort, the stratification ability of novel criteria was not so good among patients who were treated with systemic therapy. However, it is actually not advisable to include patients with R status for such analysis because the definition of R in the consensus statements is patients who are suitable for upfront surgery and those who will be benefitted from surgery itself. Therefore, inclusion of R status could be a noise and might mask important results. So, I would like to ask you whether or not there is actual survival difference when we are focusing on patients with BR1 and BR2 disease. Is there any survival difference?

Second question is about the weight of items constituting the novel oncological criteria of resectability. There are three items; size/number, presence of vascular invasion, and presence of extrahepatic disease to define the borderline resectable status. However, there has been no guarantee to handle these three items equally for prognostication. Your data suggest that if a patient has only one factor defining BR2 status, surgical resection may offer survival benefit, while among the patients who have 2–3 factors, the role of surgery is unclear. This could actually be an important result. However, we would need some rationale or evidence of handling these three items equally in statistical analysis. Given that your institution is very famous for treatment of advanced vascular invasion, I'm wondering that your population is relatively “selected” population under the institutional treatment policies. Do you have any supportive data for handling the three items rather equally? Also, I would like to ask your ideas to look for the reproducibility of data.

### A.2 Dr. Shohei Komatsu

Regarding the first question, I agree that it is not reasonable to assess the treatment outcomes of systemic chemotherapy in patients classified as R status. In this study, the treatment outcomes and characteristics of patients with the BR1 and BR2 status was also assessed. However, no statistical differences were noted between BR1 and BR2 patients in any patient characteristics, such as age, performance status, or liver function. Regarding the comparison only for the survival outcomes between BR1 and BR2, there was also no statistical difference between patients with BR1 and BR2.

As for the second question, in our cohort, patients classified in BR2 status were often associated with having several BR2 defining factors. However, when comparing the patients in BR2 obtaining only one BR2 defining factor, the prognosis tended to worse for the patients having the factor of vascular invasion than other tumor factors, including multiple tumors or extrahepatic metastases. Due to the selection bias of the patient population, it is challenging to interpretate these data, but the factor of vascular invasion may be associated with the worst potential among the three BR2 defining tumor factors. Further analysis with a large number of cases in a multicenter study is warranted.

### A.3 Dr. Shinji Itoh

Your data has very important impact on hot topic in HCC. I'd like to give one comment. Compared with patients with liver resection, patients with systemic therapy, it's relatively small, especially BR1. Further investigation using large number of patients received systemic therapy is necessary to clarify the significance of the new BR criteria in not only liver resection but also systemic therapy.

### A.4 Dr. Shohei Komatsu

I totally agree with the opinion that the number of cases for systemic chemotherapy are insufficient to investigate the actual prognostic analyses in BR criteria. Further analysis will be required to determine the utility of the BR criteria for prognostic stratification of systemic chemotherapy.
