# Supplementary material for: Treatment outcomes of hepatectomy and systemic chemotherapy based on oncological resectability criteria for hepatocellular carcinoma
Source: Ann Gastroenterol Surg. 2024 Dec 20;9(2):235–43. doi: 10.1002/ags3.12893 (PMC11877347; doi:10.1002/ags3.12893)
Supplement: Supplementary file 1 — Figure S1. [file AGS3-9-235-s002.pdf]

(A) **mALBI 1/2a**

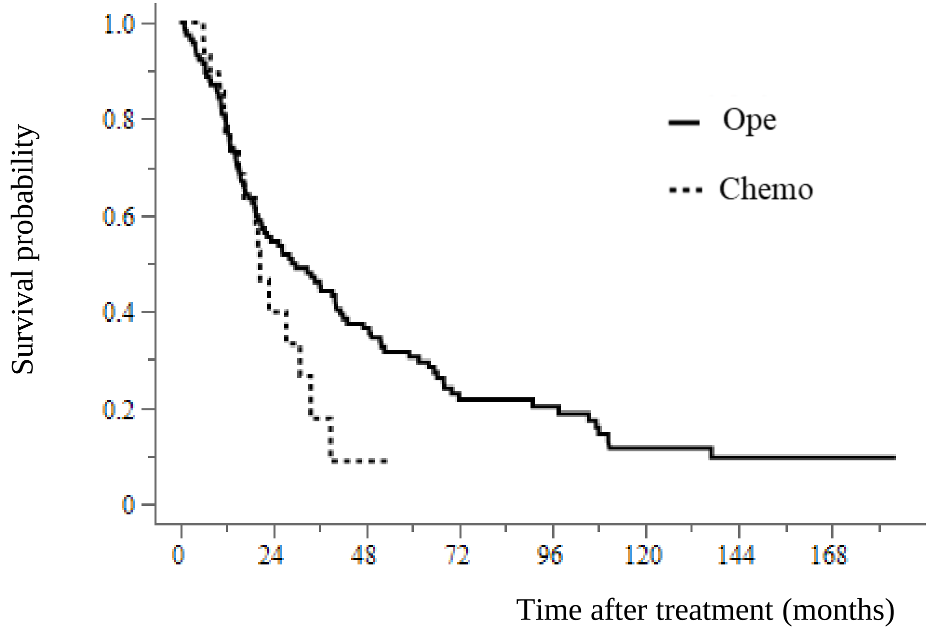

Hepatectomy (n = 118) : 28.9 month
Systemic chemotherapy (n = 33) : 20.7 month
p = 0.1652

(B) **mALBI 2b/3**

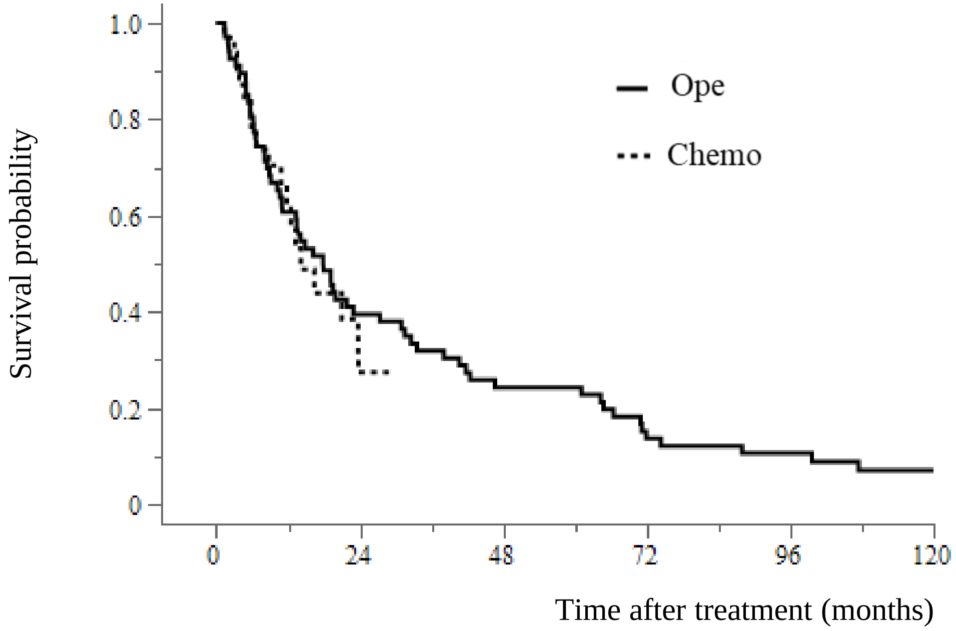

Hepatectomy (n = 68) : 18.0 month
Systemic chemotherapy (n = 34) : 14.1 month
p = 0. 5966
