# Supplementary material for: Treatment outcomes of hepatectomy and systemic chemotherapy based on oncological resectability criteria for hepatocellular carcinoma
Source: Ann Gastroenterol Surg. 2024 Dec 20;9(2):235–43. doi: 10.1002/ags3.12893 (PMC11877347; doi:10.1002/ags3.12893)
Supplement: Supplementary file 2 — Figure S2. [file AGS3-9-235-s003.pdf]

(A) **mALBI 1/2a**

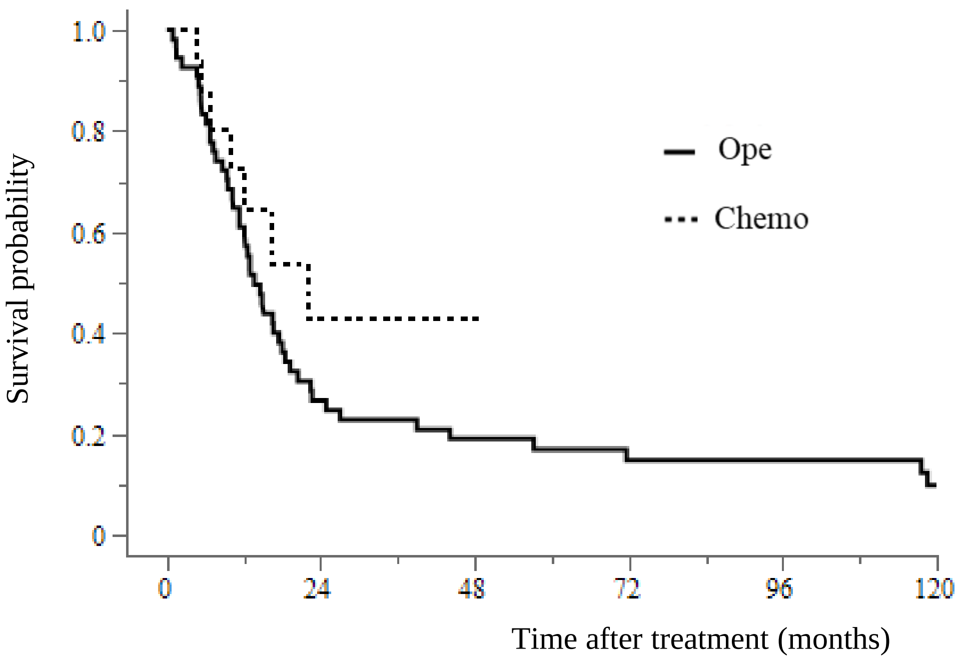

Hepatectomy (n = 54) : 13.6 month
Systemic chemotherapy (n = 16) : 21.7 month
p = 0.2464

(B) **mALBI 2b/3**

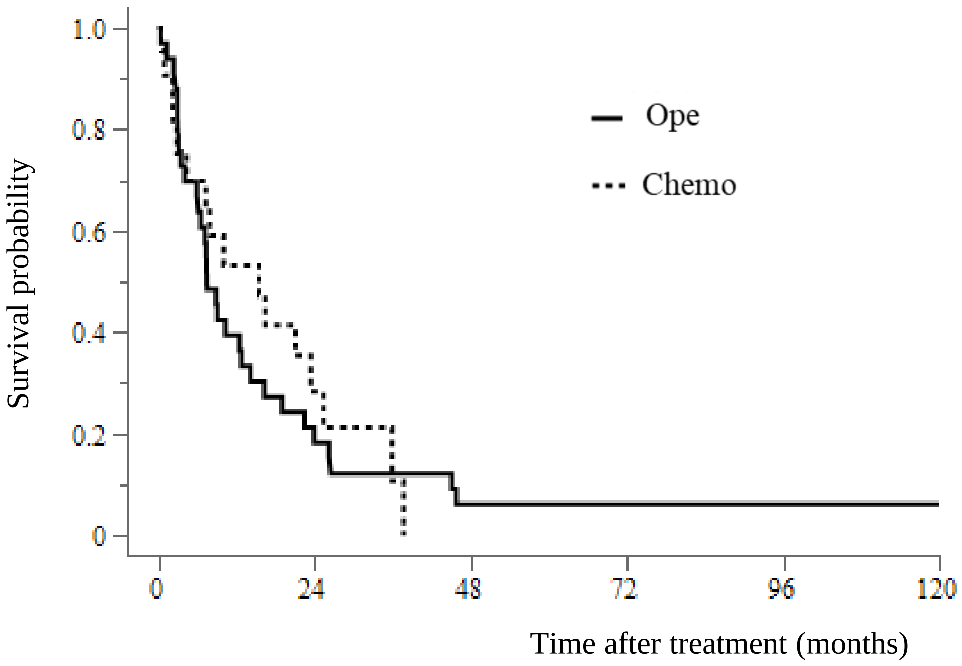

Hepatectomy (n = 33) : 7.5 month
Systemic chemotherapy (n = 22) : 15.5 month
p = 0. 6980
