# Supplementary material for: Treatment outcomes of hepatectomy and systemic chemotherapy based on oncological resectability criteria for hepatocellular carcinoma
Source: Ann Gastroenterol Surg. 2024 Dec 20;9(2):235–43. doi: 10.1002/ags3.12893 (PMC11877347; doi:10.1002/ags3.12893)
Supplement: Supplementary file 3 — Data S1. [file AGS3-9-235-s001.docx]

Figure S1: Treatment outcomes of patients with only one BR2-defining factor according to liver function (hepatectomy versus systemic chemotherapy): (A) Patients with mALBI grade 1 + 2a (B) Patients with mALBI grade 2b + 3. BR2, borderline resectable 2; mALBI: modified albumin-bilirubin.

Figure S2: Treatment outcomes of patients with two to three BR2-defining factors according to liver function (hepatectomy versus systemic chemotherapy): (A) Patients with mALBI grade 1 + 2a (B) Patients with mALBI grade 2b + 3. BR2, borderline resectable 2; mALBI: modified albumin-bilirubin.
